# A comparison of the diagnosis of gastroparesis in 4 h pediatric gastric emptying studies versus 2 h studies

**DOI:** 10.1186/s12876-019-0948-6

**Published:** 2019-02-11

**Authors:** Sarah Turnipseed Edwards, Jose Cocjin, Stephanie Bolger Theut, Douglas Rivard, Ashley K. Sherman, Craig A. Friesen

**Affiliations:** 1 Division of Gastroenterolgy, Children’s Mercy Kansas City, 2401 Gillham Rd, Kansas City, MO 64108 USA; 2 Department of Radiology, Children’s Mercy Kansas City, Kansas City, MO USA; 3 Health Services and Outcomes Research Division, Children’s Mercy Kansas City, Kansas City, MO USA; 4 Department of Pediatric Gastroenterology, Children’s Mercy Kansas City, 2401 Gillham Rd, Kansas City, MO 64108 USA

**Keywords:** Gastric emptying, Pediatrics, Scintigraphy

## Abstract

**Background:**

In adults, there is a consensus for standards to diagnose gastroparesis utilizing a gastric emptying study as the key diagnostic modality but there is no consensus for a standard in pediatrics. Additionally, some cost savings might be achieved if symptoms could be utilized to predict patients with gastroparesis. The aims of the current study were to confirm the sensitivity of a 4 h study in the pediatric population and to assess whether the severity of symptoms were predictive of delayed gastric emptying.

**Study:**

This was a single site, two part study. In the first part, results were reviewed for all patients who had completed a 4-h, solid gastric emptying study over the course of a 3 year period. In the second portion of the study, participants scheduled for a gastric emptying study, completed a modified GCSI questionnaire.

**Results:**

Out of a total of 109 participants, at 2 h, 14 participants (12.8%) had abnormal studies as compared to 26 (23.85%) participants who had abnormal studies at 4 h (*p* = .0027). Of the 95 participants with normal studies at 2 h, 15% (14/95) were abnormal at 4 h. There were no differences in symptom severity scores between those with slow and those with normal emptying at either 2 h or 4 h.

**Conclusions:**

Our study adds independent confirmation that extending studies from 2 to 4 h increases the diagnostic yield and should be the standard in children and adolescents as it is in adults.

**Electronic supplementary material:**

The online version of this article (10.1186/s12876-019-0948-6) contains supplementary material, which is available to authorized users.

## Background

Gastroparesis is delayed gastric emptying of either fluids and/or solids in the absence of mechanical obstruction. In the pediatric population, this is most commonly a post-viral process [1]. In adults, there is a consensus for standards to diagnose gastroparesis with a scintigraphic gastric emptying study being the key diagnostic modality [2]. Previously, among adult providers, the presence of multiple different methodologies for measuring gastric emptying across differing providers and institutions, including variance of performing 1, 2, 3 and 4 h gastric emptying tests, prompted a series of studies which ultimately validated the importance of 4-h studies as a more sensitive test for delayed gastric emptying and the standard for diagnosing gastroparesis [2–4].

There have been two studies in the pediatric population evaluating the possible benefits of standardized 4 h studies and the findings have been consistent with those reported in adults [5, 6]. The first was a retrospective review of 71 patients, demonstrating that 23% of patients who had normal findings at 2 h had delayed gastric emptying at 4 h [5]. The second study demonstrated an increasing positive predictive value with increasing duration of the study from 1 h to 4 h and an increased ability for 4-h studies to detect delayed emptying [6].

The evaluation of gastroparesis in children is a costly undertaking [5]. Some cost savings might be achieved if symptoms could be utilized to predict which patients have delayed gastric emptying. The Gastroparesis Cardinal Symptom index (GCSI) has been validated as a measure of symptom severity in adults with gastroparesis [7, 8]. It consists of the assessment of the severity of 9 symptoms forming 3 clusters: postprandial fullness/early satiety, nausea or vomiting, and bloating. Jericho, et al., utilized a modified GCSI in children assessing the severity of individual symptoms and found an association between delayed 4 h emptying and nausea severity [9]. Relationships to GCSI clusters were not reported.

The aims of the current study were to confirm the sensitivity of a 4 h study in the pediatric population and to assess whether the severity of individual symptoms or symptom clusters were predictive of delayed gastric emptying.

## Methods

### Study design

This was a single site, two part study comprised of both a retrospective and a prospective component. All evaluations were performed at Children’s Mercy Kansas City. This study was approved by the Institutional Review Board.

In the retrospective portion, results were reviewed for all patients who had a solid gastric emptying study performed between March 1, 2014 and March 1, 2017. Participants included those ages 2–17 years who had completed the standard 4-h study. Data collected included age at the time of study, gender, the indication for performing the study, whether the entire meal was consumed, and the 2-h and 4-h gastric emptying percentages.

The prospective study aimed to compare the results of the gastric emptying study with the data collected from the modified GCSI. In the prospective portion, patients scheduled for a gastric emptying study and fulfilling the same criteria were approached to participate in the study by completing the modified GCSI questionnaire. Gastric emptying studies were performed with the same methodology across both portions of the study. Patients were not excluded based on comorbidities or medication profile.

### Gastric emptying study

Gastric emptying studies were conducted by the standardized method, as established by Tougas and colleagues [10]. Participants were asked to fast the night prior to the gastric emptying study, for a minimum of 6 h. Participants were asked to ingest 4 oz. liquid egg white mixed with 0.5 mCi of Technetium-99 m-sulfur colloid (Cardinal Health) that had been scrambled, along with 2 slices of toasted white bread and 120 ml of water. The participants were asked to ingest the meal within 10 min. If a subject was unable to consume the entire meal the percentage of the meal consumed was noted. For the purpose of this study, we evaluated 2 h and 4 h emptying rates, which are the two time points most commonly employed in clinical practice. Anterior and posterior images were obtained using a dual head gamma camera (GE NM 630 Discovery or GE NM-CT 640 Optima) at time 0 (immediately after ingestion of meal) and at 2 h and 4 h after the meal [2]. Images were obtained using a 64 × 64 matrix with a zoom of 1 and each image was acquired for 60 s. Participants were defined as having delayed gastric emptying, based on the current adult guidelines, as follows: greater than 60% retention of gastric contents at 2 h or greater than 10% retention of gastric contents at 4 h. Severity of delayed emptying was defined as grade 1 (mild): 11–20% retention at 4 h and grade 2 (moderate): 21–35% retention at 4 h, Grade 3 (severe): 36–50% retention at 4 h, Grade 4 (very severe): > 50% retention at 4 h [2].

### Questionnaire

For the prospective study, a symptom based questionnaire [2, 4, 7, 11] was used. Two questionnaires were produced, one for parents of children aged between 2 and 12 years, who answered the questions; another questionnaire was tailored to older patients aged between 13 and 17 years (Additional files 1 and 2). Questionnaires were administered utilizing the RedCap Database. Items were adapted from the GCSI (used with permission) and used to score symptom severity for nausea, retching, vomiting, bloating, stomach fullness, loss of appetite, inability to finish a normal sized meal, ending a meal earlier due to excessive fullness and stomach visibly larger, as well as two additional questions about upper abdominal pain, lower abdominal pain. Similar to the previous pediatric study, the symptoms were scored on a 5 point Likert scale (as compared to the 6 point scale employed for adults) and ranged from 0 points for no symptoms to 4 points for very severe symptoms. Cluster scores were calculated for each of the 3 GCSI clusters as the average of symptom subscale scores within the specific cluster and included: 1. Postprandial fullness/early satiety cluster symptoms: stomach fullness, unable to finish a normal size meal, excessive fullness, and loss of appetite; 2. Nausea or vomiting cluster symptoms: nausea, vomiting, and retching; 3. Bloating cluster symptoms: bloating and stomach visibly larger. A total symptom severity score was calculated as the average of the 3 subscale scores.

### Sample size determination

We determined we needed a sample size of at least 67 pairs (2 h & 4 h data) which would have 80% power to detect a difference in proportions of 0.13 when the proportion of discordant pairs is expected to be 0.17. This is based on McNemar’s test of equality of paired proportions and uses a two-sided significance level of 0.05.

### Data analysis

For assessing the diagnostic yield of 2 h vs. 4 h studies, patients were included from both the retrospective and prospective parts of the study. For correlation of gastric emptying findings with symptoms, only data from the prospective study was analyzed.

Emptying percentages at 2 h and 4 h, respectively, were compared between patients completing the entire meal and those only partially completing the meal by the student’s t test. The percent of gastric emptying tests that were abnormal at 2 h and 4 h, respectively, were compared between patients completing the entire meal and those only partially completing the meal by chi square analysis. McNemar’s test was used to compare the proportion of patients with gastroparesis at 2 h with the proportion of patients with gastroparesis at 4 h.

The correlations between the emptying percentages at 2 h and 4 h, respectively, and the severity score for each of the 9 individual symptoms was analyzed using Spearman’s rank correlation. Mean cluster and total scores were compared between patients with normal and delayed emptying at 2 h and 4 h, respectively by the student’s t test. Correlations between emptying percentages at 2 h and 4 h, respectively, with cluster and total scores were analyzed by Pearson correlation statistics.

## Results

All patients consumed the entire egg mixture containing the Technetium-99 m-sulfur colloid (Cardinal Health). Forty-four of the 109 did not consume all of the toast and/or water. Emptying percentages did not differ between patients who completed the entire meal as opposed to those who completed a partial meal at either 2 h (62.9 ± 17.4 vs. 62.3 ± 17.4, *p* = .87) or 4 h (92.1 ± 10.7 vs. 92.6 ± 8.5, *p* = .79). Likewise, the proportion of patients with an abnormal emptying percentage did not differ between patients who completed the entire meal as opposed to those who completed a partial meal at either 2 h (12.3% vs. 13.6%, *p* = .84) or 4 h (24.6% vs. 22.7%, *p* = .86). As there were no differences between groups, all patients were included in subsequent analyses.

A total of 109 patients (64% female) were included in the analysis comparing 2 h and 4 h emptying percentages. Indications for the study are shown in Table 1. The three indications listed as “other” were written in by the participants as “not yet determined”, “residual stomach contents during EGD” and not given.

**Table 1:** Indications for gastric scintiscans

*n* = 109	*n* (%)
Age in years, mean (sd)	12.7 (3.9)
Female	70 (64)
Indication for Study	
Nausea/Vomiting	61 (56)
Abdominal pain	19 (17)
Constipation	12 (11)
GERD	10 (9)
Early satiety	2 (2)
Feeding difficulties	1 (1)
Bloating	1 (1)
Other	3 (3)

There was a significant increase in abnormal studies at 4 h, as compared to 2 h (Table 2). Of the 95 participants with normal studies at 2 h, 15% (14/95) had abnormal studies at 4 h. Of the 16 patients that were abnormal at 4 h, 69% were Grade 1, 23% were Grade 2 and 8% were Grade 4.

**Table 2:** Gastric emptying

	Normal emptying (%)	Slow emptying (%)	*p*-value
2 h	95 (87.2)	14 (12.8)	0.0027
4 h	83 (76.2)	26 (23.8)	0.0027

For the second part of the study, 38 participants completed the questionnaire. There were no differences in symptom severity scores between those with slow and those with normal emptying at either 2 h or 4 h. The mean cluster and total scores comparing those with normal and those with slow emptying at 2 h and 4 h, respectively, are shown in Tables 3 and 4. The only significant difference was that the postprandial fullness/early satiety score was elevated in patients with normal emptying at 2 h. There were no significant correlations between either 2 h or 4 h emptying percentages and any of the cluster scores or total score.

**Table 3:** Means and standard deviations (SD) for GCSI cluster and total scores for patients with normal (*n* = 30) vs. slow emptying (*n* = 8) at 2 h

Cluster	Normal emptying	Slow emptying	*P* value
Postprandial fullness/early satiety	1.88 (1.05)	1.22 (.39)	.008
Nausea or vomiting	1.36 (.81)	.88 (.85)	.15
Bloating	.92 (1.12	1.19 1.13)	.55
Total	1.48 (.66)	1.08 (.46)	.12

**Table 4:** Means and standard deviations (SD) for GCSI cluster and total scores for patients with normal (*n* = 28) vs. slow emptying (*n* = 10) at 4 h

Cluster	Normal emptying	Slow emptying	*P* value
Postprandial fullness/early satiety	1.86 (.99)	1.53 (1.00)	.37
Nausea or vomiting	1.22 (.82)	1.37 (.94)	.65
Bloating	.85 (1.13)	1.35 (1.11)	.24
Total	1.39 (.61)	1.42 (.77)	.92

We then analyzed relationships between gender, age, reasons for test with the outcome variables: emptying at 2 h and emptying at 4 h, full meal, symptom index average and the presence/absence of each of the symptoms. When assessing age, there was a significant relationship between age and full meal (*p* = 0.011). Those who completed the full meal were significantly older than those who did not complete the full meal (mean of 13.48 and sd = 3.33 vs. mean of 11.55 and sd = 4.47). When assessing reasons for the test, there were no significant relationships. When assessing gender, there were significant relationships with bloating (*p* = 0.009), upper abdomen pain (*p* = 0.019) and symptom index average (*p* = 0.005). 60.7% of the females had bloating compared to 10% of the males, 82.1% of females had upper abdomen pain compared to 40% of the males and females had a mean symptom index average of 1.57 (sd 0.58) compared to males who had a mean of 0.93 (sd 0.6).

## Discussion

This study is the third in the pediatric literature to compare the sensitivity of a 2 h and 4 h gastric emptying study in children and its ability to identify children with delayed emptying. We found that 15% of participants who had normal studies at 2 h had abnormal studies at 4 h. This is less than the 23% reported by Chogle et al., but is very similar to the findings of Wong and colleagues where 13% of patients with normal 2- h emptying were abnormal at 4 h [5, 6]. Our study adds independent confirmation that extending studies from 2 to 4 h increases the diagnostic yield and should be the standard in children and adolescents as it is in adults. In the current study, we did not find any differences in symptoms or symptom clusters between patients with delayed emptying at 4 h as compared to those with normal emptying. At 2 h, delayed emptying was actually associated with lower scores for the postprandial fullness/early satiety cluster. This might suggest that meal related symptoms are not generated by an early postprandial delay in emptying of a meal in children and adolescents. Although some studies have reported symptoms (e.g. postprandial fullness, nausea, and vomiting) associations with delayed gastric emptying of solids, there have not been any consistently reproducible relationships between specific symptoms or symptom severity and delayed emptying in adults [10, 12–16]. There have been fewer studies in children and adolescents. In pediatric patients, specific symptoms or symptom severity have generally not differed between those with delayed solid emptying as opposed to those with normal emptying [17, 18]. Utilizing 4 h studies, Wong and colleagues found no difference between those with delayed emptying as compared to those with normal emptying and in fact, within the group with delayed emptying, they reported an inverse relationship between 4 h gastric retention and nausea, vomiting, and difficulty finishing a meal, respectively [19]. In contrast, Jericho and colleagues found an association between delayed emptying and nausea severity, although there was significant overlap between those with normal and those with delayed emptying [9].

When assessing gender, there were significant relationships with bloating, upper abdomen pain and symptom index average. Females had a higher percentage of each of these symptoms, compared to the males. Although not the primary aim of the study, we evaluated children who ate the full meal as compared to those children who did not complete the entire meal. It is not uncommon for children who are being referred for a gastric emptying study to not complete the meal, as evidenced by 40% of the children not being able to complete the entire meal in our study. It is often difficult for medical providers to know how to deal with the results of a partially completed meal. Although we were not able to assess the effect of a partial meal at an individual patient level, we were able to demonstrate that it does not affect diagnostic yield at a group level. Since the gastric emptying study is based on the total amount of tracer in the stomach at the time the meal is ingested, it is perhaps not surprising that the test identifies the same percentage of patients with delayed emptying, even when they do not ingest the entire meal. It may be that the effect of toast and/or water on the emptying rate is insignificant. We did find that the older children were more likely to consume the entire meal, which could indicate the need to change the meal size depending on the age of the child, but according to our results, consumption of the entire meal, does not affect the results.

A strength of the current study is that we did not exclude patients because of co-morbid conditions or medications, which allows the results to be extrapolated more widely. Each patient served as their own control, thus we know that they were matched for co-morbid conditions and medications. This may also represent a limitation, as we did not collect this information and could not assess whether the accuracy of 2 h versus 4 h studies varies with different co-morbid conditions or medications. The current study was powered to answer the primary aim and not to assess sub-groups. Future work should address this important question.

## Conclusions

This study provides independent confirmation of previous pediatric studies at other institutions, further validating the recommendation to make 4-h studies the standard in the pediatric population. Our study also indicates that neither individual symptoms nor symptom clusters have a significant ability to identify pediatric patients with delayed solid gastric emptying. Ultimately, we think the clinical utility of gastric scintiscans will not be defined by associations with symptoms but its ability to predict responses to treatment.

## Additional files

Additional file 1: Questionnaire for ages 13–17. (DOCX 15 kb)

Additional file 2: Questionnaire for ages 2–12. (DOCX 15 kb)

## Abbreviation

GCSI: Gastroparesis Cardinal Symptom Index
