# Supplementary material for: A comparison of the diagnosis of gastroparesis in 4 h pediatric gastric emptying studies versus 2 h studies
Source: BMC Gastroenterol. 2019 Feb 11;19:26. doi: 10.1186/s12876-019-0948-6 (PMC6371451; doi:10.1186/s12876-019-0948-6)
Supplement: Supplementary file 1 — Questionnaire for ages 13–17. (DOCX 15 kb) [file 12876_2019_948_MOESM1_ESM.docx]

Questionnaire for Ages 13-17

1. Please select the best choice for symptoms experienced over the last two weeks. ?
2. Nausea: none, very mild, mild moderate, severe, very severe
3. Retching: none, very mild, mild moderate, severe, very severe
4. Vomiting: none, very mild, mild moderate, severe, very severe
5. Bloating: none, very mild, mild moderate, severe, very severe
6. Stomach Fullness: none, very mild, mild moderate, severe, very severe
7. Upper abdominal pain: none, very mild, mild moderate, severe, very severe
8. Lower abdominal pain: none, very mild, mild moderate, severe, very severe

Loss of appetite: none, very mild, mild moderate, severe, very severe

1. Unable to finish a normal sized meal: none, very mild, mild moderate, severe, very severe
2. Ending a meal earlier due to excessively full: none, very mild, mild moderate, severe, very severe
3. Stomach visibly larger: none, very mild, mild moderate, severe, very severe
4. Do you have diabetes?

Did you measure your glucose this morning before the gastric emptying test? Yes No

If yes, what was the value?

1. Have you taken any pain medication in the past two days? (including Percocet, percodan, Demerol, Tylenol #3, oxycodone,methadone, and morphine). Yes No

If yes, which one?

How often do you take this?

When did you last take this type of medicine?

1. Do you take any medications to speed up your GI tract (stomach or colon) (including Reglan,erythromycin, and bethanchol. Yes No
2. List any other medicine you currently take:
3. Have you had any surgeries on your GI tract – the esophagus, stomach or colon? Yes No
4. Please circle the number that most accurately describes the majority of your stools:

Bristol Stool Form Scale [4]

Type

1 Separate hard lumps, like nuts

2 Sausage shaped but lumpy

3 Like a sausage or snake but with cracks on its surface

4 Like a sausage or snake, smooth and soft

5 Soft blobs with clear cut edges

6 Fluffy pieces with ragged edges, a mushy stool

7 Watery, no solid pieces
