# Supplementary material for: A comparison of the diagnosis of gastroparesis in 4 h pediatric gastric emptying studies versus 2 h studies
Source: BMC Gastroenterol. 2019 Feb 11;19:26. doi: 10.1186/s12876-019-0948-6 (PMC6371451; doi:10.1186/s12876-019-0948-6)
Supplement: Supplementary file 2 — Questionnaire for ages 2–12. (DOCX 15 kb) [file 12876_2019_948_MOESM2_ESM.docx]

Questionnaire for Ages 2-12

1. Please select the best choice for symptoms experienced over the last two weeks.
2. Nausea: none, very mild, mild moderate, severe, very severe
3. Retching: none, very mild, mild moderate, severe, very severe
4. Vomiting: none, very mild, mild moderate, severe, very severe
5. Bloating: none, very mild, mild moderate, severe, very severe
6. Stomach Fullness: none, very mild, mild moderate, severe, very severe
7. Upper abdominal pain: none, very mild, mild moderate, severe, very severe
8. Lower abdominal pain: none, very mild, mild moderate, severe, very severe

Loss of appetite: none, very mild, mild moderate, severe, very severe

1. Unable to finish a normal sized meal: none, very mild, mild moderate, severe, very severe
2. Ending a meal earlier due to excessively full: none, very mild, mild moderate, severe, very severe
3. Stomach visibly larger: none, very mild, mild moderate, severe, very severe
4. Does your child have diabetes?

If yes, did you measure your child’s glucose this morning before the gastric emptying test? Yes No

If yes, what was the value?

1. Has your child taken any pain medication the past two days? These including Percocet, percodan, Demerol, Tylenol #3, oxycodone, morphine and methadone. Yes No

If yes, which one?

How often does your child take this medication?

When did your child last take this type of medicine?

1. Does your child take any medications to speed up their GI tract (stomach or colon)? including Reglan, erythromycin and bethanchol. Yes No
2. Please, list any other medicine your child is currently taking:
3. Has your child had any surgeries on their GI tract – the esophagus, stomach or colon? Yes No
4. Please circle the number that most accurately describes the majority of your child’s stools:

Bristol Stool Form Scale

Type

1 Separate hard lumps, like nuts

2 Sausage shaped but lumpy

3 Like a sausage or snake but with cracks on its surface

4 Like a sausage or snake, smooth and soft

5 Soft blobs with clear cut edges

6 Fluffy pieces with ragged edges, a mushy stool

7 Watery, no solid pieces
